# 

**Published:** 1893-09

**Authors:** 


### CONTENTS FOR SEPTEMBER, 1893.

PAGE.

ORIGINAL COMMUNICATIONS.

Intra-uterine Asphyxia, with Report of Three Cases. By GEO. F. HULBERT, M. D. ... 65
Double Synchronous Amputation of Both Legs in an Infant—Recovery. By GREGORY DOYLE, M. D. ... 74
Uterine Fibroids: Some Facts in Regard to these Neoplasms. By HENRY D. INGRAHAM, M. D. ... 75
How Shall We Treat Scarlet Fever? By FREDERIC W. BARTLETT, M. D. ... 78
Concerning Posture. By B. H. DAGGETT, M. D. ... 85

PROGRESS IN MEDICAL SCIENCE.

Surgery ... 89

SELECTIONS.

Three Ships with Beriberi Outbreaks ... 99
Summer Diseases ... 105

ABSTRACTS.

Ear Cough ... 106
Spinal Concussion ... 107
Three Cases of Pertussis Treated with Bromoform ... 108
Scope and Limitations of Cerebral Surgery ... 108
Bow-legs ... 109
The Action of Glycerine in Nephrolithiasis ... 110

EDITORIAL.

Advanced Medical Education ... 111
The Need for Greater Uniformity in the Division of the Abdominal Regions ... 113
Topics of the Month ... 114
Personal ... 116
Academy of Medicine Notes ... 116
College Notes ... 117
Society Meetings ... 118
Book Reviews ... 123
Miscellany ... 126

### Index to Advertisements.

PAGE.

PAGE.

